# Improved Individualized Patient-Oriented Depth-of-Hypnosis Measurement Based on Bispectral Index

**DOI:** 10.3390/s23010293

**Published:** 2022-12-27

**Authors:** Gorazd Karer, Igor Škrjanc

**Affiliations:** Faculty of Electrical Engineering, University of Ljubljana, 1000 Ljubljana, Slovenia

**Keywords:** general anesthesia, total intravenous anesthesia, target-controlled infusion, propofol, BIS index, depth of hypnosis, improved mathematical model, population-data-based model, residual model

## Abstract

Total intravenous anesthesia is an anesthesiologic technique where all substances are injected intravenously. The main task of the anesthesiologist is to assess the depth of anesthesia, or, more specifically, the depth of hypnosis (DoH), and accordingly adjust the dose of intravenous anesthetic agents. However, it is not possible to directly measure the anesthetic agent concentrations or the DoH, so the anesthesiologist must rely on various vital signs and EEG-based measurements, such as the bispectral (BIS) index. The ability to better measure DoH is directly applicable in clinical practice—it improves the anesthesiologist’s assessment of the patient state regarding anesthetic agent concentrations and, consequently, the effects, as well as provides the basis for closed-loop control algorithms. This article introduces a novel structure for modeling DoH, which employs a residual dynamic model. The improved model can take into account the patient’s individual sensitivity to the anesthetic agent, which is not the case when using the available population-data-based models. The improved model was tested using real clinical data. The results show that the predictions of the BIS-index trajectory were improved considerably. The proposed model thus seems to provide a good basis for a more patient-oriented individualized assessment of DoH, which should lead to better administration methods that will relieve the anesthesiologist’s workload and will benefit the patient by providing improved safety, individualized treatment, and, thus, alleviation of possible adverse effects during and after surgery.

## 1. Introduction

Adequate general anesthesia (GA) is a prerequisite in surgeries as well as in various other medical procedures. The anesthesiologist must take care of three main aspects of the patient state during the procedure: besides considering the vital signs, they must administer substances that keep the patient deeply unconscious, prevent the patient from feeling pain, and keep the patient’s muscles adequately relaxed. Furthermore, the patient must not be aware of, or even remember, what was happening during GA. Therefore, it is essential to properly administer the needed substances during the medical procedure.

GA and the dynamic response of the patient’s body to anesthetic substances can be regarded as quite complex dynamic systems. Various pharmacokinetic (PK) and pharmacodynamic (PD) mechanisms take place inside the patient’s body; however, it is unfortunately generally not yet possible to claim that these PK and PD systems have been completely studied or adequately modeled. Furthermore, the anesthetic depth is a controversial concept, which involves the three main aspects of hypnosis, analgesia, and muscle relaxation. In addition, amnesia must also be ensured. However, anesthetic depth cannot be directly measured.

The main job of the anesthesiologist is to monitor the patient’s vital functions and properly maintain the functions of the patient’s organs. Anesthetic agents are administered in various ways into the patient’s body so as to achieve adequate GA: the two most commonly used methods in clinical practice are the intravenous administration induction of the anesthetic agent, i.e., injection of the anesthetic agent into a patient’s vein, or the inhalatory administration of anesthetic agent, in which the substance is induced by the patient inhaling a prepared breathing mixture. If the anesthetic agents are injected intravenously, the anesthesiologic technique is known as *total intravenous anesthesia* (TIVA) [1,2]. In this article, we focus on TIVA administration exclusively.

During the medical procedure, the anesthesiologist aims to maintain the appropriate depth of hypnosis (DoH) by adjusting the dosage of the anesthetic agent. Clearly, the PK and PD of the anesthetic agent and the type of procedure must be taken into account, e.g., long-term sedation in an intensive care unit requires deeper DoH than GA during surgery. Too-deep anesthesia is manifested as a drop in blood pressure level and heart rate frequency as well as slow post-operative awakening of the patient from GA [3]. On the other hand, the opposite is also true. Moreover, inadequate depth of anesthesia results in the activation of sympathetic nerves, or, in the most unlikely event, with the patient awakening, which must be avoided at all costs [4]. In modern clinical practice, DoH is determined by assessing the relevant clinical signs (iris, sweating, movements), by interpreting hemodynamic measurements [5] and by estimating the DoH from EEG signals [6]. The latter is made possible by several established measurement systems, such as BIS index, NeuroSense, Narcotrend, Entropy (Scale and Response), WAV${}_{CNS}$, and Patient State Index.

BIS index is a noninvasive measurement method. A BIS monitor is connected to electrodes on the patient’s head and the bispectral index is calculated from the measured EEG signals. The BIS monitor provides a single dimensionless number, which ranges from 0 (equivalent to EEG silence) to 100. A BIS value between 40 and 60 indicates an appropriate level for GA, whereas a value below 40 is appropriate for long-term sedation due to head injuries. The reference can thus be set to the applicable value; the manner and speed of approaching the reference value depend on the specific characteristics of the procedure and the pharmacokinetics and pharmacodynamics of the substance in the patient’s body. The BIS value can, therefore, be considered as a representation of the DoH, although some papers question the relevance of BIS measurement for representing hypnosis as a graded state during surgery [7,8]. The authors in [7] pointed out the poor correlation between BIS and serum concentrations of propofol, which calls into question the relevance of the BIS measurement in representing hypnosis as a graded state during surgery. When they included all values during anesthesia, there was a significant correlation between BIS and serum propofol, but the correlation was poor and disappeared when the outliers were removed. Their results do not seem to support the idea that BIS represents a continuous measure of DoH, assuming that elevated serum concentrations of propofol correspond to deeper hypnotic levels. However, because they have too few data points to test the correlation between serum concentration and BIS in individual patients, the reason could be that there are interindividual differences in sensitivity to propofol. They noted that a correlation could exist in a single patient but is not evident in data from many subjects.

In the literature, there are several approaches to modeling the effect of propofol, which is a hypnotic anesthetic agent. For this purpose, a number of PK and PD models have been developed, e.g., [9,10,11,12]. The models of propofol response define the general structure of the dynamic system, whereas the particular parameters depend on the individual patient’s characteristics, such as gender, age, height, weight, etc., as well as the particular patient’s individual sensitivity to the effects of propofol and their ability to have propofol eliminated from the body. Certain infusion pumps employ the PK models to enable *target controlled infusion* (TCI), where the pump sets the proper flow of the medication with regard to the model [13].

In the last years, an emerging paradigm in medicine seems to have grasped the idea of personalized medicine [14]. In the field of drug delivery systems, this includes modeling, control, analysis, and pharmacological studies, as well as development of novel medical devices and conducting of targeted clinical trials. In this regard, the systematic employment of dynamic-system analysis along with control theory offers a wide range of application opportunities in the medical domain [15,16,17,18,19,20]. Despite the fact that for TCI various PK models can be implemented, all having their own advantages and drawbacks, the Marsh [21] and Schnieder [22,23] models are mainly used in clinical practice. However, these models often do not reflect the actual dynamic properties that depend on individual sensitivity to the substance of the particular patient, which is generally not considered. For instance, the authors in [24] found that titration of propofol based on BIS monitoring allows a reduction in drug consumption that is associated with a similar decrease in propofol plasma concentrations compared with TCI. An inverse relationship between cardiac output and plasma propofol concentration was reported in [25]. In addition, the authors in [26] showed that remifentanil plasma concentrations during remifentanil and propofol anesthesia are influenced by cardiac output in a similar manner to propofol, although the metabolic sites are different. Because cardiac output is known not to be constant during anesthesia, this also significantly affects plasma concentrations and, thus, the effects of propofol and remifentanil. The authors concluded that actual blood concentrations of remifentanil and propofol may differ significantly from expected concentrations, especially when cardiac output is low. Cardiac output is therefore an important factor whose influence should be investigated in PK and PD models. This again supports the idea that individualized PK and PD modeling could improve anesthetic delivery methods. Therefore, approaches using population-based-data models often cannot ensure optimal performance, especially when treating a patient with a particularly considerable discrepancy from the population-data-based models. A population-data-based approach cannot yield universally usable models, even if a very large number of patients would be taken into account and despite the fact that only relatively healthy patients are considered.

The dynamic mathematical models can be directly used in clinical practice for assessing DoH-related variables by implementing them in soft sensors [27] or state observers [28], which would improve the anesthesiologist’s assessment of the patient state regarding anesthetic agent concentrations and, consequently, the effects, hence benefiting the patients in the long run. In addition, the models can enable in silico testing of various potentially clinically implementable closed-loop control algorithms, e.g., PID-controller-based approaches [29,30,31]. Furthermore, they represent the basis for a number of advanced control approaches, such asrobust control [32,33,34], model-predictive control [35,36], fuzzy-rule-based decision system [37], event-based control [38], etc. Despite difficulties in objective pain measurement [18,39], a number of articles also consider the inherent MIMO (or MISO) nature of the controlled system, which is due to drug interactions, especially when analgesics (such as remeifentanil) are considered [40,41,42].

The article introduces a novel structure for modeling DoH. The modeling framework results in an improved individualized assessment of DoH, which is reflected in better predictions of the related anesthetic agent concentrations as well as the measured BIS signal. The article is structured as follows. First, we introduce the basic three-compartmental model and the upgraded population-data-based model, namely, the PK and PD parts. Next, we introduce the improved model structure and present the residual dynamic model add-on. In Section 4, the identification procedure is presented, including the identification signals, filtering, and parameter estimation. This is further extended to online recursive parameter estimation. Next, we validate the improved model by comparing the simulated data for the particular patient to real clinically acquired data, which were logged during a surgery in a real clinical setting. We end the article with some concluding remarks.

## 2. Propofol Pharmacokinetic and Pharmacodynamic Modeling

The pharmacokinetics of the basic population-data-based model is described by the Schnider model [22,23]. A well-established three-compartmental model structure is used as the basis for dynamic relations. For details, see [43,44]. The internal dynamics of the model can be formulated using Equations (1)–(3).

(1) $$\begin{array}{c} \frac{dx_{1}}{dt}=\phi -k_{12}x_{1}-k_{13}x_{1}-k_{10}x_{1}+k_{21}x_{2}+k_{31}x_{3} \end{array}$$

(2) $$\begin{array}{c} \frac{dx_{2}}{dt}=-k_{21}x_{2}+k_{12}x_{1} \end{array}$$

(3) $$\begin{array}{c} \frac{dx_{3}}{dt}=-k_{31}x_{3}+k_{13}x_{1} \end{array}$$

In Equations (1)–(3), the variables $x_{1}$, $x_{2}$, and $x_{3}$ represent the amount of the drug in compartments $V_{1}$, $V_{2}$, and $V_{3}$, respectively. The infusion flow rate is denoted as ϕ. As noted above, the parameters $k_{12}$, $k_{21}$, $k_{13}$, and $k_{31}$ represent the partition coefficients that determine the speed at which the drug moves from one particular compartment to another. Finally, $k_{10}$ is the rate of elimination of the drug from the patient’s body. Note that the concentration in the central compartment is often referred to as plasmatic concentration.

The effect site for the drug propofol is basically the central nervous system. The effect site is thus part of the central compartment, but the effect of the drug is subject to some dynamics with regard to the (theoretical) concentration in the central compartment [44]. Therefore, a first-order model was used to describe the effect-site concentration dynamics, as given in Equation (4).

(4) $$\begin{array}{c} \frac{dx_{e}}{dt}=-k_{e0}x_{e}+k_{e0}x_{1} \end{array}$$

The effect-site concentration of propofol is considered as the main influence on the DoH. Despite acknowledging that DoH is a multivariable and a not very easy to grasp concept, involving deep unconsciousness, analgesia, amnesia, and muscle relaxation, we want to keep the model as simple as possible, yet not too simple for our requirements. Therefore, we first assume that BIS index is an adequate measure for DoH, despite the fact that the assumption might be debatable [8,45,46]. Furthermore, we consider an SISO model, where the input represents propofol inflow, and the output represents the value of the BIS index.

Despite the fact that the PD effect mechanism has not been fully studied yet, in the literature, a sigmoid $E_{max}$ model based on the general Hill equation [47] is usually considered, as given in Equation (5).

(5) $$\begin{array}{cc} y_{BIS} & =y_{BIS}\left(x_{e}\right)\\ y_{BIS} & =BIS_{0}-\left(BIS_{0}-BIS_{min}\right)\frac{x_{e}^{\gamma }}{x_{e50}^{\gamma }+x_{e}^{\gamma }} \end{array}$$

In Equation (5), $BIS_{0}$ denotes the characteristic value for a fully awake patient, $BIS_{min}$ stands for the minimum value of BIS index, and γ is the parameter that defines the nonlinear shape of the response curve.

Therefore, the combined model can be regarded structurally as a Wiener nonlinear model.

The parameters of the phamacokinetic model are taken from [22,23,48]. The values are given in Table 1.

Note that the values of model parameters depend on the patient’s age, weight, height, and gender. Parameter LBM is calculated as given in Equation (6).

(6) $$\begin{array}{cc} \; & LBM=\\ \; & \left\{\begin{array}{cc} 1.1\cdot weight/\mathrm{kg}-128{\left(\frac{weight/\mathrm{kg}}{height/\mathrm{cm}}\right)}^{2} & ;\mathrm{male}\\ 1.07\cdot weight/\mathrm{kg}-148{\left(\frac{weight/\mathrm{kg}}{height/\mathrm{cm}}\right)}^{2} & ;\mathrm{female} \end{array}\right. \end{array}$$

The parameters of the BIS-index-effect output submodel (see Equation (5)) are set as suggested in [49] and are given in Table 2.

## 3. Residual Model Introduction—Improvement of the Population-Data-Based Model

Population-data-based mathematical models, such as the one described in the previous section, are regularly used in clinical practice, namely, for TCI propofol infusion, which has become a standard approach in administration of the anesthetic agent. In spite of this, we need to consider the fact that the available models are derived from population-based measurements, both regarding the pharmacokinetic and the pharmacodynamic part.

Prior to a medical procedure, the implemented mathematical model is always tuned to the particular patient’s properties (e.g., age, weight, height, and gender). Despite this, the model is still based on a broader population sample that was involved in the measured-data gathering. Hence, it is impossible for such a model to take into account specific interpatient variabilities. Therefore, the basic population-data-based model can broadly predict DoH, but it can exhibit severe discrepancies from the actual BIS-indicated DoH of a particular patient, especially when the particular patient’s sensitivity to the anesthetic agent is considerably different from the dynamics assumed in the mathematical model.

**Figure 1:**
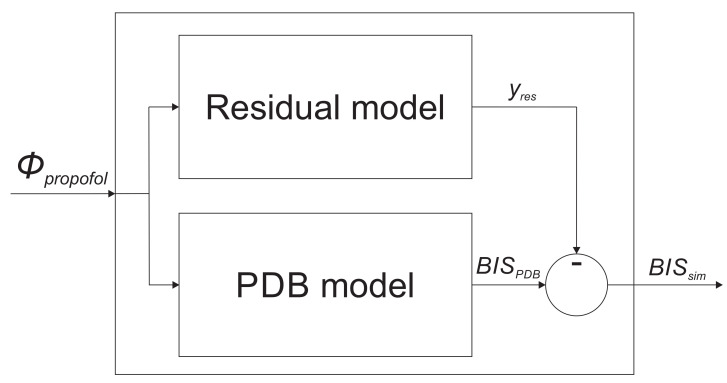
The improved model structure consisting of the population-data-based (PBD) model, and the residual dynamic model.

In order to improve the population-data-based model accuracy by taking into account the specific patient’s individual dynamic properties, we propose an extension to the basic model structure. The whole model is then structured as follows: besides the population-data-based model, we introduce an additional residual model, which is intended to mathematically describe the dynamic discrepancy of the patient’s particular sensitivity to anesthetic-agent infusion. The discrepancy signal represents the ideal residual model output. The improved model structure, with the additional residual dynamic add-on, is shown in Figure 1. The output of the improved model $BIS_{sim}$ is, therefore, calculated as given in Equation (7).

(7) $$\begin{array}{c} BIS_{sim}=BIS_{PDB}-y_{res} \end{array}$$

Here, $BIS_{PDB}$ denotes the basic population-data-based model output, and $y_{res}$ is the residual model output.

The goal of introducing the residual model is thus to improve the combined model output. This means that the combined model includes the population-data-based model as the basis for modeling the patient dynamic response to propofol. In addition, the residual model considers the particular patient’s individual dynamic response to propofol, namely, the individual patient discrepancies from the population-data-based model, thus enabling a more accurate assessment of DoH expressed by BIS index. Note that the following conditions prevent a patient from being included in the study: patients with poor general condition (ASA > 3), patients with BMI > 35, drug addicts, patients taking psychotropic medicines or opioid analgesics (including tramadol), patients with a severe psychiatric disease or central nervous system disease (except the reason for surgery), patients with arrhythmia affecting or preventing the measurements (e.g., chronic atrial fibrillation), and patients that received benzodiazepines.

## 4. Identification of the Residual Dynamic Model

### 4.1. Identification Signals

The residual dynamic model is identified based on the data measured during a medical procedure treating an individual patient. The input of the residual dynamic model is the actual propofol inflow $\phi_{propofol}$, whereas its output is obtained by subtracting the actual measured BIS signal $BIS_{data}$ from the population-data-based model output $BIS_{PDB}$, as shown in Equation (8). The latter is calculated according to the predefined population-data-based PK and PD model, taking into account the individual patient’s properties.

Both signals are sampled using $T_{s}=1\;s$ sampling rate. The whole experiment is therefore represented by two discrete-time signals in Equations (8) and (9).

(8) $$\begin{array}{c} y_{res,id}\left(z\right)=BIS_{PDB}\left(z\right)-BIS_{data}\left(z\right) \end{array}$$

(9) $$\begin{array}{c} u_{res,id}\left(z\right)=\phi_{propofol}\left(z\right) \end{array}$$

### 4.2. Residual Dynamic Model Structure

The structure of the residual dynamic model should be as simple as possible, but at the same time complex enough to be able to adequately model the patient’s discrepancy from the population-data-based model. Despite that higher complexity of the models and possibly evolving identification could yield favorable approximations [50,51,52], especially if the identification data are noise-free, we established that an appropriate structure for our case is a second-order model, which is structured as an affine autoregressive model with exogenous inputs. Its discrete-time formulation is given in Equation (10).

(10) $$\begin{array}{c} y_{res}\left(t+T_{s}\right)=a_{1}y_{res}\left(t\right)+a_{2}y_{res}\left(t-T_{s}\right)+b_{1}u_{res}\left(t\right)+c \end{array}$$

Here, $a_{1}$, $a_{2}$, $b_{1}$, and *c* represent the parameters to be estimated. Note that *t* represents a particular time-instant of the model. A single step of the model $T_{s}$ is not necessarily equal to the data-sampling rate $T_{s,data}$. In our case, it is $T_{s}=5\cdot T_{s,data}=5\;s$.

The model parameters can be gathered in the parameter vector Θ as given in Equation (11).

(11) $$\begin{array}{c} \Theta^{T}=\left[a_{1},a_{2},b_{1},c\right] \end{array}$$

The regressor is defined in Equation (12).

(12) $$\begin{array}{c} \Psi {\left(t\right)}^{T}=\left[y_{res}\left(t\right),y_{res}\left(t-T_{s}\right),u_{res}\left(t\right),1\right] \end{array}$$

In this way, Equation (10) can be rewritten in Equation (13).

(13) $$\begin{array}{c} y_{res}\left(t+T_{s}\right)=\Theta^{T}\Psi \left(t\right) \end{array}$$

### 4.3. Parameter Estimation

The parameters of the residual dynamic model $a_{1}$, $a_{2}$, $b_{1}$, and *c* are estimated from the measured data concerning the particular patient.

As the measured $BIS_{data}$ signal is prone to significant noise, the first step is to apply a suitable filter, which should ensure better identification results. We established that a simple first-order filter (with a filtering time-constant $\tau_{f}$) is adequate. The filter can be represented by the transfer function in Equation (14). Its discrete-time equivalent is given in Equation (15).

(14) $$\begin{array}{cc} \tau_{f} & =20\;s\\ H_{f}\left(s\right) & =\frac{1}{\tau_{f}s+1}=\frac{1}{20s+1} \end{array}$$

(15) $$\begin{array}{c} H_{f}\left(z\right)=\frac{0.04877}{z-0.9512} \end{array}$$

Finally, the filtered identification signals are given in Equations (16) and (17).

(16) $$\begin{array}{c} y_{res,id,f}\left(z\right)=H_{f}\left(z\right)y_{res,id}\left(z\right) \end{array}$$

(17) $$\begin{array}{c} u_{res,id,f}\left(z\right)=H_{f}\left(z\right)u_{res,id}\left(z\right) \end{array}$$

The filtered signals $y_{res,id,f}$ and $u_{res,id,f}$ are used for estimating the parameters of the residual dynamic model Θ.

The output data vector Y contains the output variable as given in Equation (18).

(18) $$\begin{array}{c} Y=\left[\begin{array}{c} y_{res,id,f}\left(t_{1}\right)\\ \vdots \\ y_{res,id,f}\left(t_{P}\right) \end{array}\right] \end{array}$$

The regression matrix Ψ is obtained by using the whole set of measured data, as given in Equations (19) and (20).

(19) $$\begin{array}{c} \Psi =\left[\begin{array}{c} \Psi_{f}^{T}\left(t_{1}\right)\\ \vdots \\ \Psi_{f}^{T}\left(t_{P}\right) \end{array}\right] \end{array}$$

(20) $$\begin{array}{c} \Psi {\left(t_{i}\right)}_{f}^{T}=\left[y_{res,id,f}\left(t_{i}\right),y_{res,id,f}\left(t_{i}-T_{s}\right),u_{res,id,f}\left(t_{i}\right),1\right] \end{array}$$

Here, $t_{i}$ represents a time instant concerning a particular identification data pair (i=1,…,P).

According to Equations (18)–(20) and (13), the parameters of the residual dynamic model Θ can be obtained using the least-squares identification method, as given in Equation (21).

(21) $$\begin{array}{c} \Theta ={\left(\Psi^{T}\Psi \right)}^{-1}\Psi^{T}Y \end{array}$$

## 5. Online Recursive Parameter Estimation

In order to implement the proposed modeling framework as an intelligent soft sensor for assessing DoH, we must be able to acquire the improved combined model for DoH during surgery. The population-data-based submodel is based on patient data and can be derived prior to surgery. On the other hand, the particular patient’s individual dynamic response to propofol, namely, the individual patient discrepancies from the population-data-based model, can only be assessed after the measured data becomes available, i.e., either after finishing the medical procedure, as proposed in Section 4.3, which can rarely be used, or during the medical procedure as soon as new data become available. The latter approach enables the use of the improved model during the medical procedure.

The recursive parameter estimation is carried out as described below. The residual model in Equations (10) and (13) is linear in the parameters, therefore it is possible to analytically derive a least-squares estimate of the parameters. Furthermore, if the identified system parametersare expected to be time-varying, the online estimation algorithm can place more emphasis on newly acquired data and gradually discard older data. Therefore, the proposed approach implements a recursive least-squares identification with exponential forgetting [53]. The algorithm can consider a least-squares loss function for exponentially discarding older data as time passes. The model parameters are, therefore, estimated using Equations (22)–(24).

(22) $$\begin{array}{c} \hat{\Theta }\left(t\right)=\hat{\Theta }\left(t-T_{s}\right)+K\left(t\right)\left(y_{res,id,f}\left(t\right)-\Psi_{f}^{T}\left(t\right)\hat{\Theta }\left(t-T_{s}\right)\right) \end{array}$$

(23) $$\begin{array}{c} K\left(t\right)=P\left(t-T_{s}\right)\Psi_{f}\left(t\right){\left(\lambda +\Psi_{f}^{T}\left(t\right)P\left(t-T_{s}\right)\Psi_{f}\left(t\right)\right)}^{-1} \end{array}$$

(24) $$P\left(t\right)=\left(I-K\left(t\right)\Psi_{f}^{T}\left(t\right)\right)P\left(t-T_{s}\right)/\lambda$$

In Equations (22)–(24), P(t) denotes the covariance matrix ($P\left(t\right)\in R^{n\times n}$), where *n* represents the length of the regressor. $\hat{\Theta }\left(t\right)$ denotes the vector of the identified or estimated process parameters, λ denotes the forgetting factor, and *I* is the unity matrix, where $I\in R^{n\times n}$. The recursive parameter estimation is carried out online—in every time step *t*—and returns the calculated parameters of the model $\hat{\Theta }\left(t\right)$.

The forgetting factor λ is defined in Equation (25), where $t_{\lambda }$ stands for the time constant for the exponential forgetting and $T_{s}$ is the sampling time.

(25) $$\lambda =e^{-\frac{T_{s}}{t_{\lambda }}}$$

The regressive parameter estimation method can consider time-varying dynamics of the identified process; therefore, exponential forgetting can be employed. The forgetting factor has to be set between 0<λ≤1. In this manner, the data used for parameter estimation are pondered, so that the last data are pondered by factor 1, whereas the data that are $k_{s}\cdot T_{s}$ time steps old are pondered only by a factor of $\lambda^{k_{s}}$.

The initial covariance matrix P(0) has to be positive-definite and properly sized. If the regressive parameter estimation method is interpreted as a Kalman filter, it can be regarded as ensuring that the parameters are distributed with an initial covariance P(0) and initial mean-values $\hat{\Theta }\left(0\right)$ [53].

In each time step *t*, a new estimation is calculated. The new parameter estimations $\hat{\Theta }\left(t\right)$ are recursively based on the estimations from the previous time step $t-T_{s}$ and the online newly measured data. Therefore, the resulting model adapts to the new measurements as soon as they are available.

## 6. Model Validation Based on Real Clinical Data

In order to validate the proposed modeling approach, we gathered real data during a TIVA medical procedure. In our case, the treated patient was a 46-year-old male who weighed 59 kg and was 175 cm tall.

### 6.1. Recorded Signals

**Figure 2:**
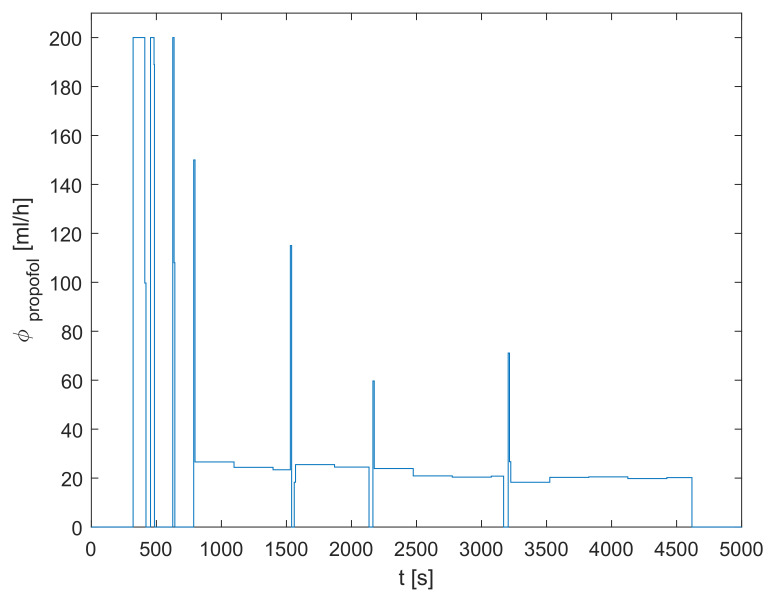
The inflow of propofol $\phi_{propofol}$.

The signal denoting the inflow of propofol $\phi_{propofol}$ was parsed from the clinically recorded data and is shown in Figure 2. One can see that, firstly, a bolus dose is introduced in order to rapidly increase the concentration of propofol in the body. This phase is called the induction of anesthesia and results in the patient losing consciousness. Later, a suitable dose of propofol is continuously administered in order to keep the proper anesthetic depth.

**Figure 3:**
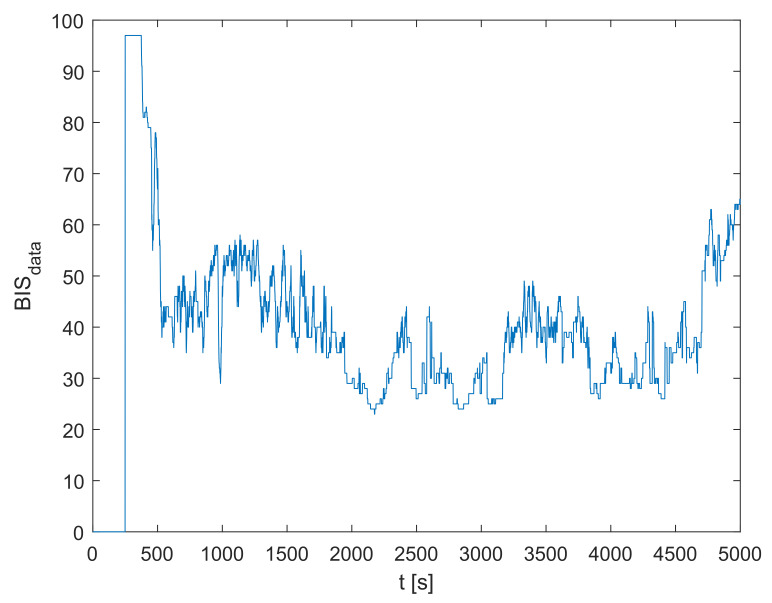
The measured BIS trajectory $BIS_{data}$.

**Figure 4:**
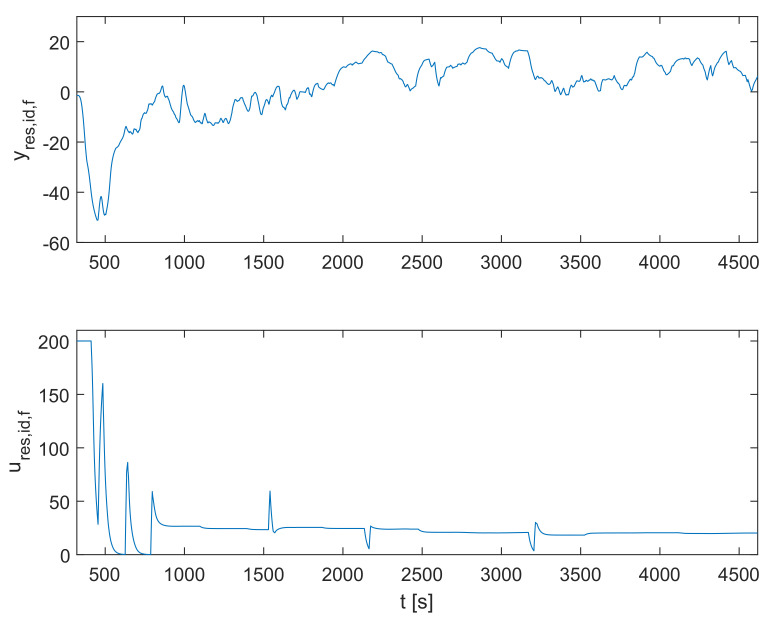
The identification signals for the residual dynamic model $y_{res,id,f}$ and $u_{res,id,f}$.

The recorded $BIS_{data}$ signal for the particular treated patient is shown in Figure 3. The plasmatic concentration of propofol $c_{p}$ and effect-site concentration of propofol $c_{e}$ are obtained by simulating the dynamic model defined in Equations (1)–(4) based on the measured inflow signal of propofol $\phi_{propofol}$. Next, the population-data-based BIS trajectory $BIS_{PDB}$ is obtained according to Equation (5). Finally, it is possible to obtain the identification signals for the residual dynamic model $y_{res,id,f}$ and $u_{res,id,f}$ using Equations (8), (9), and (15)–(17). The identification signals are shown in Figure 4. Note that only the relevant interval of the measured data is considered.

### 6.2. Identification Results

The final resulting parameters of the residual dynamic model are given in Equation (26).

(26) $$\begin{array}{c} \Theta^{T}=\left[1.7376,-0.7469,-0.0053,0.1638\right] \end{array}$$

When conducting online recursive parameter estimation, the parameters converge to the values in Equation (26). Before starting, the initial values for the algorithm are set as given in Equations (27)–(29).

(27) $$\begin{array}{c} P\left(0\right)=100\cdot I=\left[\begin{array}{cccc} 100 & 0 & 0 & 0\\ 0 & 100 & 0 & 0\\ 0 & 0 & 100 & 0\\ 0 & 0 & 0 & 100 \end{array}\right] \end{array}$$

(28) $$\begin{array}{c} {\hat{\Theta }}^{T}\left(0\right)=\left[1.7242,-0.7356,-0.0040,0.1729\right] \end{array}$$

(29) $$\begin{array}{c} \lambda =1 \end{array}$$

**Figure 5:**
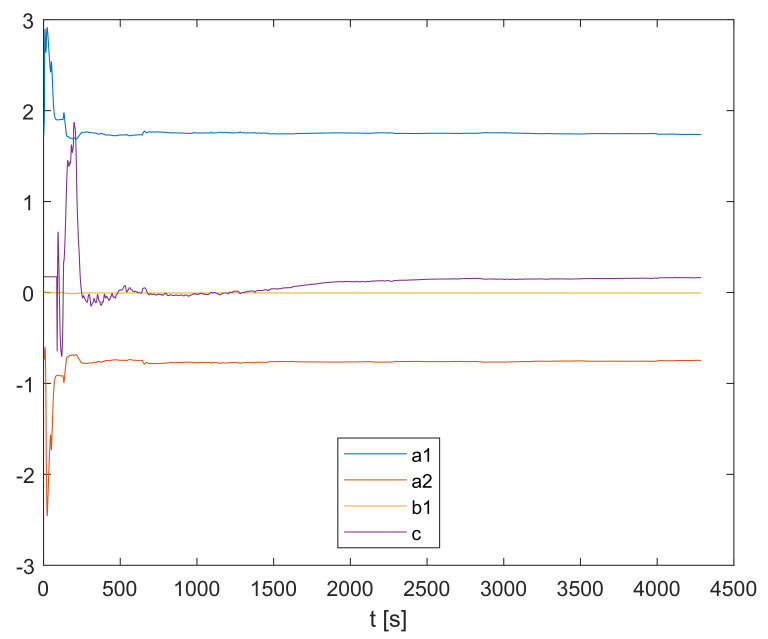
Online recursive parameter estimation.

As the recursive parameter estimation is carried out, the parameters $a_{1},a_{2},b_{1}$, and *c* change their values along the trajectories shown in Figure 5.

### 6.3. Model Validation

In order to validate the obtained model, we employ a quantitative measure for predictive quality assessment, which is often used in the literature, namely, the *prediction mean square error* (PMSE). It is calculated according to Equation (30).

(30) $$\begin{array}{c} PMSE_{x}=\frac{1}{N}\sum_{i=1}^{N}{\left(x_{i,sim}-x_{i,data}\right)}^{2} \end{array}$$

Furthermore, there are two more quantitative measures that are often used in the literature to compare the simulated and the measured signal, such as the BIS-index trajectory: the *median performance error* (MDPE), which is calculated according to Equation (31), and *median absolute performance error* (MDAPE), which is calculated according to Equation (32).

(31) $$\begin{array}{c} MDPE_{x}=median{\left\{\frac{x_{i,data}-x_{i,sim}}{x_{i,sim}}\cdot 100\%\right\}}_{i=1,\ldots ,N} \end{array}$$

(32) $$\begin{array}{c} MDAPE_{x}=median\{|\frac{x_{i,data}-x_{i,sim}}{x_{i,sim}}{\left|\cdot 100\%\right\}}_{i=1,\ldots ,N} \end{array}$$

In Equations (30)–(32), $x_{sim}$ and $x_{data}$ denote the simulated and the measured data, respectively, whereas *N* stands for the number of data points in the dataset.

**Figure 6:**
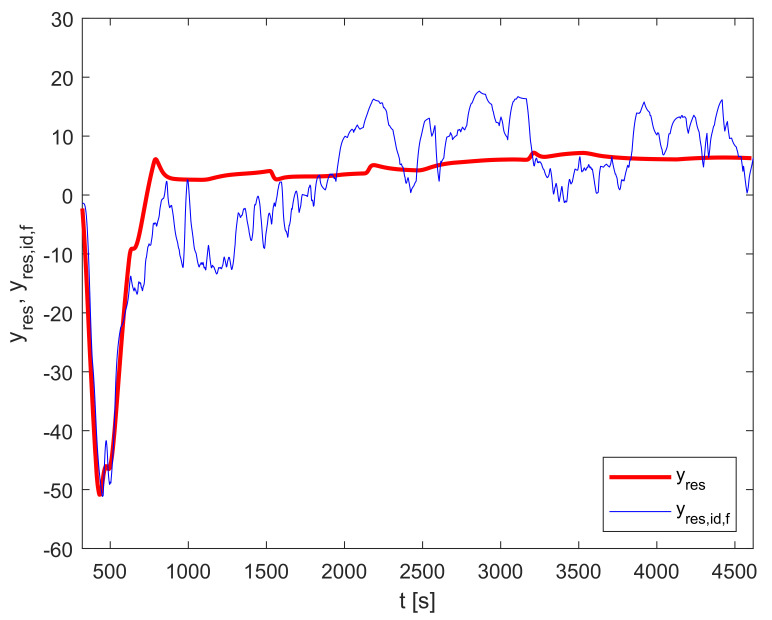
Residual dynamic model validation: $y_{res}$ and $y_{res,id,f}$.

Figure 6 shows the output of the residual dynamic model $y_{res}$ compared to the measurements-based trajectory $y_{res,id,f}$.

**Figure 7:**
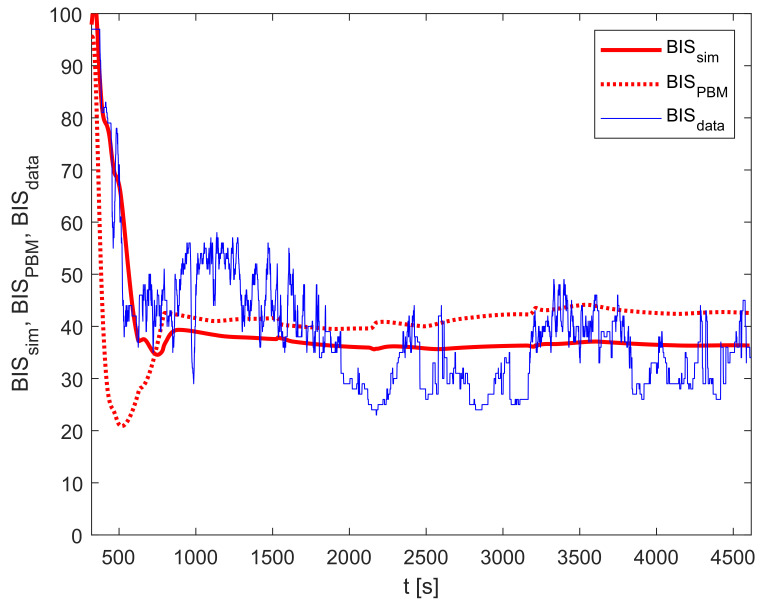
Improved model validation: $BIS_{sim}$, $BIS_{PBM}$, and $BIS_{data}$.

Finally, the output of the improved model is calculated according to Equation (7). The relevant BIS trajectories are shown in Figure 7.

For the case of the particular patient and medical procedure, the calculated criteria for $y_{res}$ and BIS are given in Table 3 and Table 4, respectively.

The presented residual dynamic model reduces the PMSE of the difference between $y_{res}$ and $y_{res,id,f}$ from 166.0 to 50.8; therefore, the improvement factor is 3.27.

The implementation of the improved model reduces the PMSE from 179.8 ($BIS_{BMS}$ and $BIS_{data}$) to 63.3 (for $BIS_{sim}$ and $BIS_{data}$). In this case, the improvement factor is 2.84. Furthermore, the MDPE criterion improved by 10.24%, and MDAPE by 5.64%.

The main limitation of the proposed model is that it cannot be rigorously validated for a specific patient before its implementation because of its dependence on online measurements collected during the medical procedure. Moreover, no two anesthetic applications are alike, even if the same patient undergoes the same type of surgery several times, for example. Since the dynamic characteristics of the patient may change over time, it is not possible to compare the performance in two different interventions even if the same patient is treated. Because we cannot claim that the individualized dynamic model in question is time-invariant in the long run, it cannot be validated by using a special validation dataset that is strictly separate from the dataset used for identification. Furthermore, as the goal of the additional residual model is to consider the individual patient’s discrepancy from the population-data-based model, it is not sensible to cross-validate it using another patient’s measurements.

On the other hand, the results suggest that a significant improvement can be achieved, compared with the approach based only on the population-data-based model. In the future, the modeling framework will be further verified with several more datasets on different individuals treated with TIVA.

## 7. Conclusions

The article introduces a novel structure for modeling DoH, resulting in an improved individualized assessment of DoH, which is reflected in better predictions of the measured BIS signal. The presented model structure for modeling DoH dynamics employs the residual dynamic model add-on, which is used to model a particular treated patient’s individual dynamic discrepancies from the population-data-based model. In such a manner, the model can take into account the patient’s individual sensitivity to the anesthetic agent, which is not the case when using the population-data-based model exclusively, e.g., when using TCI, as is often the case in clinical practice. Therefore, the proposed modeling framework provides an improved mechanism for predicting DoH measured by the BIS index.

The improved model was verified using real clinical data logged during a medical treatment of a particular patient that lasted a little more than one hour. The results show that the predictions of the BIS-index trajectory were, indeed, considerably improved. Hence, the improved model seems to provide a solid foundation for better simulations as well as for the implementation in closed-loop model-based predictive control of DoH. The modeling framework will be further verified with several more datasets concerning various individuals that have been treated with TIVA.

To sum up, the presented framework provides a basis for a more patient-oriented individualized model for assessing DoH. The model seems to provide a deeper insight into DoH dynamics, which should lead to better administration methods that will relieve the anesthesiologist’s workload and will benefit the patient by providing improved safety, individualized treatment, and, thus, alleviation of possible adverse effects during and after surgery.

## Figures and Tables

**Table 1:** Parameter values.

Parameter	Value
$V_{c}$	4.27l
$k_{10}$	0.443 + 0.0107·(weight/kg − 77) − − 0.0159·(LBM/kg − 59) + + 0.0062·(height/cm − 177)/min
$k_{12}$	0.302 − 0.0056·(age/years − 53)/min
$k_{13}$	0.196/min
$k_{21}$	[1.29 − 0.024·(age/years − 53)] · ·[18.9 − 0.391·(age − 53)]${}^{-1}$/min
$k_{31}$	0.0035/min
$k_{e0}$	0.456/min

**Table 2:** Parameter values.

Parameter	Value
$BIS_{0}$	95.6
$BIS_{min}$	8.9
$x_{e50}$	2.23
γ	1.58

**Table 3:** Predictive quality measures for $y_{res}$.

$y_{\mathrm{res}}$	PMSE	MDPE	MDAPE
Without residual model	166.0	N/A	N/A
With residual model	50.8	−4.88	101.4
Improvement	3.27	N/A	N/A

**Table 4:** Predictive quality measures for BIS.

BIS	PMSE	MDPE	MDAPE
Without residual model	179.8	−10.85%	22.56%
With residual model	63.3	−0.605%	16.92%
Improvement	2.84	10.24%	5.64%

## Data Availability

The data used are available upon request to the corresponding author.

## References

[1] Al-Rifai Z., Mulvey D. (2016). Principles of total intravenous anaesthesia: Practical aspects of using total intravenous anaesthesia. BJA Educ..

[2] Absalom A.R., Glen J.B., Zwart G.J., Schnider T.W., Struys M.M. (2016). Target-Controlled Infusion: A Mature Technology. Anesth. Analg..

[3] Wesselink E.M., Kappen T.H., Torn H.M., Slooter A.J., van Klei W.A. (2018). Intraoperative hypotension and the risk of postoperative adverse outcomes: A systematic review. Br. J. Anaesth..

[4] Tasbihgou S.R., Vogels M.F., Absalom A.R. (2018). Accidental awareness during general anaesthesia–A narrative review. Anaesthesia.

[5] Potočnik I., Janković V.N., Štupnik T., Kremžar B. (2011). Haemodynamic changes after induction of anaesthesia with sevoflurane vs. propofol. Signa Vitae.

[6] Musizza B., Ribaric S. (2010). Monitoring the Depth of Anaesthesia. Sensors.

[7] Hoymork S.C., Raeder J., Grimsmo B., Steen P.A. (2003). Bispectral index, serum drug concentrations and emergence associated with individually adjusted target-controlled infusions of remifentanil and propofol for laparoscopic surgery. Br. J. Anaesth..

[8] Sleigh J.W. (2011). Depth of AnesthesiaPerhaps the Patient Isn’t a Submarine. Anesthesiology.

[9] Kataria B.K., Ved S.A., Nicodemus H.F., Hoy G.R., Lea D., Dubois M.Y., Mandema J.W., Shafer S.L. (1994). The pharmacokinetics of propofol in children using three different data analysis approaches. Anesthesiology.

[10] Schüttler J., Ihmsen H. (2000). Population pharmacokinetics of propofol: A multicenter study. Anesthesiology.

[11] Kenny G.N., White M. (1990). Intravenous propofol anaesthesia using a computerised infusion system. Anaesthesia.

[12] Eleveld D.J., Colin P., Absalom A.R., Struys M.M. (2018). Pharmacokinetic–pharmacodynamic model for propofol for broad application in anaesthesia and sedation. Br. J. Anaesth..

[13] Neckebroek M., Ionescu C.M., van Amsterdam K., Smet T.D., Baets P.D., Decruyenaere J., Keyser R.D., Struys M.M. (2019). A comparison of propofol-to-BIS post-operative intensive care sedation by means of target controlled infusion, Bayesian-based and predictive control methods: An observational, open-label pilot study. J. Clin. Monit. Comput..

[14] Ionescu C.M., Neckebroek M., Ghita M., Copot D. (2021). An Open Source Patient Simulator for Design and Evaluation of Computer Based Multiple Drug Dosing Control for Anesthetic and Hemodynamic Variables. IEEE Access.

[15] Brogi E., Cyr S., Kazan R., Giunta F., Hemmerling T.M. (2017). Clinical Performance and Safety of Closed-Loop Systems. Anesth. Analg..

[16] Zaouter C., Joosten A., Rinehart J., Struys M.M., Hemmerling T.M. (2020). Autonomous systems in anesthesia: Where do we stand in 2020? A narrative review. Anesth. Analg..

[17] Pasin L., Nardelli P., Pintaudi M., Greco M., Zambon M., Cabrini L., Zangrillo A. (2017). Closed-loop delivery systems versus manually controlled administration of total IV Anesthesia: A meta-analysis of randomized clinical trials. Anesth. Analg..

[18] Ghita M., Neckebroek M., Juchem J., Copot D., Muresan C.I., Ionescu C.M. (2020). Bioimpedance Sensor and Methodology for Acute Pain Monitoring. Sensors.

[19] Puri G.D., Kumar B., Aveek J. (2007). Closed-loop anaesthesia delivery system (CLADS™) using bispectral index: A performance assessment study. Anaesth. Intensive Care.

[20] Liu N., Chazot T., Trillat B., Pirracchio R., Law-Koune J.D., Barvais L., Fischler M. (2006). Feasibility of closed-loop titration of propofol guided by the Bispectral Index for general anaesthesia induction: A prospective randomized study. Eur. J. Anaesthesiol..

[21] Marsh B., White M., Morton N., Kenny G.N. (1991). Pharmacokinetic model driven infusion of propofol in children. Br. J. Anaesth..

[22] Schnider T.W., Minto C.F., Gambus P.L., Andresen C., Goodale D.B., Shafer S.L., Youngs E.J. (1998). The influence of method of administration and covariates on the pharmacokinetics of propofol in adult volunteers. Anesthesiology.

[23] Schnider T.W., Minto C.F., Shafer S.L., Gambus P.L., Andresen C., Goodale D.B., Youngs E.J. (1999). The influence of age on propofol pharmacodynamics. Anesthesiology.

[24] Bauer M., Wilhelm W., Kraemer T., Kreuer S., Brandt A., Adams H.A., Hoff G., Larsen R. (2004). Impact of bispectral index monitoring on stress response and propofol consumption in patients undergoing coronary artery bypass surgery. Anesthesiology.

[25] Kurita T., Morita K., Kazama T., Sato S. (2002). Influence of cardiac output on plasma propofol concentrations during constant infusion in swine. Anesthesiology.

[26] Kurita T., Uraoka M., Jiang Q., Suzuki M., Morishima Y., Morita K., Sato S. (2013). Influence of cardiac output on the pseudo-steady state remifentanil and propofol concentrations in swine. Acta Anaesthesiol. Scand..

[27] Blažič A., Škrjanc I., Logar V. (2021). Soft sensor of bath temperature in an electric arc furnace based on a data-driven Takagi–Sugeno fuzzy model. Appl. Soft Comput..

[28] Nogueira F.N., Mendonça T., Rocha P. (2019). Positive state observer for the automatic control of the depth of anesthesia—Clinical results. Comput. Methods Programs Biomed..

[29] Schiavo M., Padula F., Latronico N., Paltenghi M., Visioli A. (2022). A modified PID-based control scheme for depth-of-hypnosis control: Design and experimental results. Comput. Methods Programs Biomed..

[30] Padula F., Ionescu C., Latronico N., Paltenghi M., Visioli A., Vivacqua G. (2017). Optimized PID control of depth of hypnosis in anesthesia. Comput. Methods Programs Biomed..

[31] Gonzalez-Cava J.M., Carlson F.B., Troeng O., Cervin A., van Heusden K., Dumont G.A., Soltesz K. (2021). Robust PID control of propofol anaesthesia: Uncertainty limits performance, not PID structure. Comput. Methods Programs Biomed..

[32] Hosseinzadeh M. (2020). Robust control applications in biomedical engineering: Control of depth of hypnosis. Control. Appl. Biomed. Eng. Syst..

[33] Dumont G.A., Martinez A., Ansermino J.M. (2009). Robust control of depth of anesthesia. Int. J. Adapt. Control. Signal Process..

[34] Heusden K.V., Dumont G.A., Soltesz K., Petersen C.L., Umedaly A., West N., Ansermino J.M. (2014). Design and clinical evaluation of robust PID control of propofol anesthesia in children. IEEE Trans. Control. Syst. Technol..

[35] Sawaguchi Y., Furutani E., Shirakami G., Araki M., Fukuda K. (2008). A model-predictive hypnosis control system under total intravenous anesthesia. IEEE Trans. Biomed. Eng..

[36] Pawłowski A., Schiavo M., Latronico N., Paltenghi M., Visioli A. (2022). Model predictive control using MISO approach for drug co-administration in anesthesia. J. Process. Control.

[37] Mendez J.A., Leon A., Marrero A., Gonzalez-Cava J.M., Reboso J.A., Estevez J.I., Gomez-Gonzalez J.F. (2018). Improving the anesthetic process by a fuzzy rule based medical decision system. Artif. Intell. Med..

[38] Merigo L., Beschi M., Padula F., Latronico N., Paltenghi M., Visioli A. (2017). Event-Based control of depth of hypnosis in anesthesia. Comput. Methods Programs Biomed..

[39] Neckebroek M., Ghita M., Ghita M., Copot D., Ionescu C.M. (2020). Pain Detection with Bioimpedance Methodology from 3-Dimensional Exploration of Nociception in a Postoperative Observational Trial. J. Clin. Med..

[40] Janda M., Schubert A., Bajorat J., Hofmockel R., Nöldge-Schomburg G.F., Lampe B.P., Simanski O. (2013). Design and implementation of a control system reflecting the level of analgesia during general anesthesia. Biomed. Tech..

[41] Hemmerling T.M., Arbeid E., Wehbe M., Cyr S., Taddei R., Zaouter C., Reilly C.S. (2013). Evaluation of a novel closed-loop total intravenous anaesthesia drug delivery system: A randomized controlled trial. Br. J. Anaesth..

[42] Peñaranda C.C., Arroyave F.D.C., Gómez F.J., Corredor P.A.P., Fernández J.M., Botero M.V., Bedoya J.D.B., Toro C.M. (2020). Technical and clinical evaluation of a closed loop TIVA system with SEDLineTM spectral density monitoring: Multicentric prospective cohort study. Perioper. Med..

[43] Karer G. (2018). Modelling of Target-Controlled Infusion of Propofol for Depth-of-Anaesthesia Simulation in Matlab-Simulink. Proceedings of The 9th EUROSIM Congress on Modelling and Simulation, EUROSIM 2016, The 57th SIMS Conference on Simulation and Modelling SIMS 2016.

[44] Karer G., Novak-Jankovič V., Stecher A., Potočnik I. (2018). Modelling of BIS-Index Dynamics for Total Intravenous Anesthesia Simulation in Matlab-Simulink. IFAC PapersOnLine.

[45] Ni K., Cooter M., Gupta D.K., Thomas J., Hopkins T.J., Miller T.E., James M.L., Kertai M.D., Berger M. (2019). Paradox of age: Older patients receive higher age-adjusted minimum alveolar concentration fractions of volatile anaesthetics yet display higher bispectral index values. Br. J. Anaesth..

[46] Kreuzer M., Stern M.A., Hight D., Berger S., Schneider G., Sleigh J.W., Garciá P.S. (2020). Spectral and Entropic Features Are Altered by Age in the Electroencephalogram in Patients under Sevoflurane Anesthesia. Anesthesiology.

[47] Goutelle S., Maurin M., Rougier F., Barbaut X., Bourguignon L., Ducher M., Maire P. (2008). The Hill equation: A review of its capabilities in pharmacological modelling. Fundam. Clin. Pharmacol..

[48] Operator’s Guide: Infusion Workstation: Orchestra Base Primea. https://www.google.com.hk/url?sa=t&rct=j&q=&esrc=s&source=web&cd=&ved=2ahUKEwiUuIrb1Jb8AhWmr1YBHV7oAjAQFnoECAkQAQ&url=http%3A%2F%2Fwww.frankshospitalworkshop.com%2Fequipment%2Fdocuments%2Finfusion_pumps%2Fuser_manuals%2FFresenius%2520Orchestra%2520Base%2520Unit%2520-%2520User%2520manual.pdf&usg=AOvVaw0-StEgOXitUKevQvWEwzsx.

[49] Martín-Mateos I., Méndez J.A., Reboso J.A., Leõn A. (2013). Modelling propofol pharmacodynamics using BIS-guided anaesthesia. Anaesthesia.

[50] Andonovski G., Lughofer E., Skrjanc I. (2021). Evolving Fuzzy Model Identification of Nonlinear Wiener-Hammerstein Processes. IEEE Access.

[51] Škrjanc I., Andonovski G., Iglesias J.A., Sesmero M.P., Sanchis A. (2022). Evolving Gaussian on-line clustering in social network analysis. Expert Syst. Appl..

[52] Ožbot M., Lughofer E., Škrjanc I. (2022). Evolving Neuro-Fuzzy Systems based Design of Experiments in Process Identification. IEEE J. Mag.|IEEE Xplore.

[53] Åström K.J., Wittenmark B. (1995). Adaptive Control.

